# Clinical, Radiological, Pathological Features and Seizure Outcome With Surgical Management of Polymorphous Low-Grade Neuroepithelial Tumor of the Young Associated With Epilepsy

**DOI:** 10.3389/fonc.2022.863373

**Published:** 2022-03-18

**Authors:** Xiaorui Fei, Jing Zhao, Wei Wei, Wei Wang, Xue Kong, Ruobing Qian, Chaoshi Niu, Yang Yao

**Affiliations:** ^1^ Department of Neurosurgery, The First Affiliated Hospital of USTC, Division of Life Sciences and Medicine, University of Science and Technology of China, Hefei, China; ^2^ Department of Radiology, The First Affiliated Hospital of USTC, Division of Life Sciences and Medicine, University of Science and Technology of China, Hefei, China; ^3^ Department of Pathology, The First Affiliated Hospital of USTC, Division of Life Sciences and Medicine, University of Science and Technology of China, Hefei, China; ^4^ Intelligent Pathology Institute, Division of Life Sciences and Medicine, University of Science and Technology of China, Hefei, China

**Keywords:** polymorphous low-grade neuroepithelial tumor of the young, epilepsy, surgical effect, pathology, radiology, genetic alterations

## Abstract

**Objective:**

Polymorphous low-grade neuroepithelial tumor of the young (PLNTY) is a novel distinct epileptogenic neoplasm, and its clinical, imaging, histopathological, and molecular features were already known in the existing literature. We aimed to analyze the surgical management of PLNTY combined with these known characteristics.

**Methods:**

Eight patients underwent surgical treatment in our center between December 2017 and December 2020, and the postoperative pathology was diagnosed as PLNTY. Their clinical data, imaging, pathological, molecular characteristics, and seizure outcome were retrospectively analyzed. Follow-up evaluations and a literature review were performed.

**Results:**

The 8 patients included 1 woman and 7 men, aged between 5 and 51 years old (mean = 31.6, median = 29). The preoperative symptoms of all 8 cases were seizures. Four tumors were situated in the temporal lobes, and one of the four extratemporal tumors was in the occipital lobe and three were in the frontal lobe. Enlarged and gross total resections were performed in 2 cases and the other 6 cases, respectively. All cases exhibited intense labeling of CD34, and absence of 1p/19q codeletion and IDH1 or IDH2 mutation. B-Raf proto-oncogene (BRAF) V600E mutation was presented in 4 (66.7%) of 6 detected cases. The postoperative seizure outcome of Engel class I was achieved in 6 cases (75%).

**Conclusion:**

PLNTY represents distinctive histologic, immunophenotypic and biomolecular features, and has high epileptogenicity. Early surgical intervention and enlarged resection of PLNTY associated with epilepsy will help to improve the postoperative seizure-free rate.

## Introduction

Polymorphous low-grade neuroepithelial tumor of the young (PLNTY), a rare variant of the low-grade neuroepithelial tumor, was first described in 2017 by Huse et al. (1). Till now, only approximately 55 cases of PLNTY have been reported (1–14). Similar to ganglioglioma (GG), oligodendroglioma, pleomorphic xanthoastrocytoma (PXA), and dysembryoplastic neuroepithelial tumor (DNET), PLNTY was also defined as a member of long-term epilepsy-associated brain tumors (LEATs) or epileptomas (15, 16). However, PLNTY has distinct clinical, radiological, pathological, and molecular characteristics when compared to other kinds of LEATs.

PLNTY is often encountered in children and young adults, involving the temporal lobe (17–19). As for radiological features, PLNTY is often morphologically presented as a well-circumscribed lesion, solid with a frequent cystic alteration. In most of the cases, the CT shows a single lesion presented as intraparenchymal calcification, and the MRI appears as iso- or low signal in T1WI and high signal in T2WI, and with slight or no enhancement (4). Pathologically, the most evident morphological feature of PLNTY is infiltrative growth pattern with a mass of oligodendroglioma-like cellular components, and intense immunohistochemical staining with CD34. Recently, the molecular pathological characteristics of PLNTY have been reported as genetic alteration of BRAF V600E or fibroblast growth factor receptors 2 and 3 (FGFR2, FGFR3), which may be related to the pathogenesis of PLNTY (1).

PLNTY was included as a newly recognized tumor type in the 2021 WHO classification of tumors of the central nervous system, and was assigned to WHO grade I (16). However, it exhibits a strong epileptogenic propensity. Most PLNTY cases were found with intracranial lesions following new-onset seizures, some were found after a long history of refractory epilepsy. Previous reports have proved the seizure-free effect and optimal prognosis following surgical resection. However, not all PLNTY patients are free of seizure onset. The factors associated with the seizure control effect remain unclear. In this study, we will report a series of 8 PLNTY cases in our center and review the literature, aiming to explore surgical treatment and clinical management of PLNTY combined with its known common characteristics.

## Methods

### Clinical Data

This study retrospectively reviewed the medical records, surgical procedures, radiological, pathological, and molecular data of 8 cases with PLNTY who underwent surgery at our center from December 2017 to December 2020. Individualized presurgical evaluations, namely, history, symptoms, brain CT and MRI, detailed evaluation of seizure semiology, and long-term video-EEG monitoring, were performed in all patients. The MRI examinations used sequences, namely, conventional T1WI and T2WI sequence, T2 fluid-attenuated inversion recovery (T2 Flair) sequence, and T1WI after gadolinium enhancement. The radiological examination was reviewed by two independent neuroradiologists.

### Surgical Procedures

All surgical plans were individually tailored. Preoperative video-EEG monitoring of epileptiform discharges in ictal or interictal periods was used to confirm the correlation between the occurrence of seizure and tumor. 3D image processing based on MRI to display the precise location of the tumor, adjacent structures of the tumor, and the shape of gyrus, aimed to plan a suitable surgical approach and resection range to increase the total tumor resection rate. Furthermore, MR-based neuronavigation and intraoperative electrocorticography (ECoG) were used during surgery. The tumor resection range was categorized as gross total resection (GTR, no distinct residual tumor), subtotal resection (STR, >90% of tumor removal), partial resection (PTR, <90% of tumor removal), and enlarged resection (ER). The enlarged resection included resection of tumors and peripheral cortex with abnormal discharge, which was conducted with the guide of intraoperative ECoG during surgery. Intraoperative ECoG identified abnormal spiking outside the tumor margin, and abnormal discharges remained in the peritumoral cortex after gross tumor resection, which guided us to perform ER.

### Pathological Examination and Immunohistochemistry

The surgically resected tissues were fixed in paraffin with formaldehyde and stained with hematoxylin–eosin to observe their microscopic appearance. The streptavidin–biotin peroxidase complex method was used to conduct the immunohistochemical studies included the following antibodies: glial fibrillary acidic protein (GFAP), CD34, oligodendrocytic transcription factor 2 (Olig-2), soluble protein-100 (S100), synaptophysin (Syn), neurofilament (NF), neuronal nuclear antigen (NeuN), P53, Ki-67, α-thalassemia mental retardation X-linked (ATRX), and mutant isocitrate dehydrogenase 1/2 (IDH1/2). The pathological examination was performed by two experienced neuropathologists.

### Molecular Examination

The genomic DNA of the tumors was isolated from paraffin-embedded tissue samples. The 1p/19q deletion was detected by fluorescent *in situ* hybridization (FISH), and the real-time PCR technique was utilized for detecting BRAF V600E gene mutation, using AmoyDx BRAF V600 Mutations Detection Kit and Applied Biosystems 7500 Real-Time PCR (Thermo Fisher Scientific). The internal control VIC signal of the tested samples should have an obvious amplification curve, and the Ct value should be between 13 and 21. If the FAM signal has an obvious amplification curve and the Ct value is less than 28, the sample does not need to be repeated, and the test result is positive for the BRAF V600E mutation; if the Ct value is ≥28, the test result is negative for the BRAF V600E mutation.

### Follow-Up

The follow-up was conducted in the outpatient department every three months. Post-operative brain MRI was recommended to monitor the recurrence of the tumor, and seizure outcome was graded according to the Engel Outcome Scale (20): Engel class I (seizure-free with or without AEDs), Engel class II (nearly seizure free, ≤2 times per year), Engel class III (seizures significantly reduced, more than 90% reduction), and Engel class IV (no reduction or aggravation). Engel class I was defined as a good prognosis, while Engel class II–IV was defined as a poor prognosis.

### Literature Review

The literature review was conducted by the search of the English literature related to PLNTY in PubMed up to December 2021. The keywords used in the search were (“polymorphous low-grade neuroepithelial tumor”) and (“PLNTY”). The cases with clinical data provided and pathologically proven as PLNTY were included. While, the papers were excluded when the full text was unavailable, or the cases reported by the papers had incomplete clinical data or were not pathologically determined as PLNTY.

## Results

### Patient Demographics

This study included 8 patients (seven men and one woman) with a mean age of 31.6 years (range between 5 and 51 years) at surgery, and 3 patients were older than 30 years. The preoperative symptoms were all partial epilepsy, and the duration of epilepsy ranged from 2 to 50 months, with a mean length of 17.4 months. Among them, 1 case (case a) received two or more first-line antiepileptic drugs (AEDs) treatment regularly, 3 cases received one AED and 4 cases were not formally treated with AEDs. No patient had a history of previous radiotherapy or surgery.

### Radiological Evaluation

**Figure 1:**
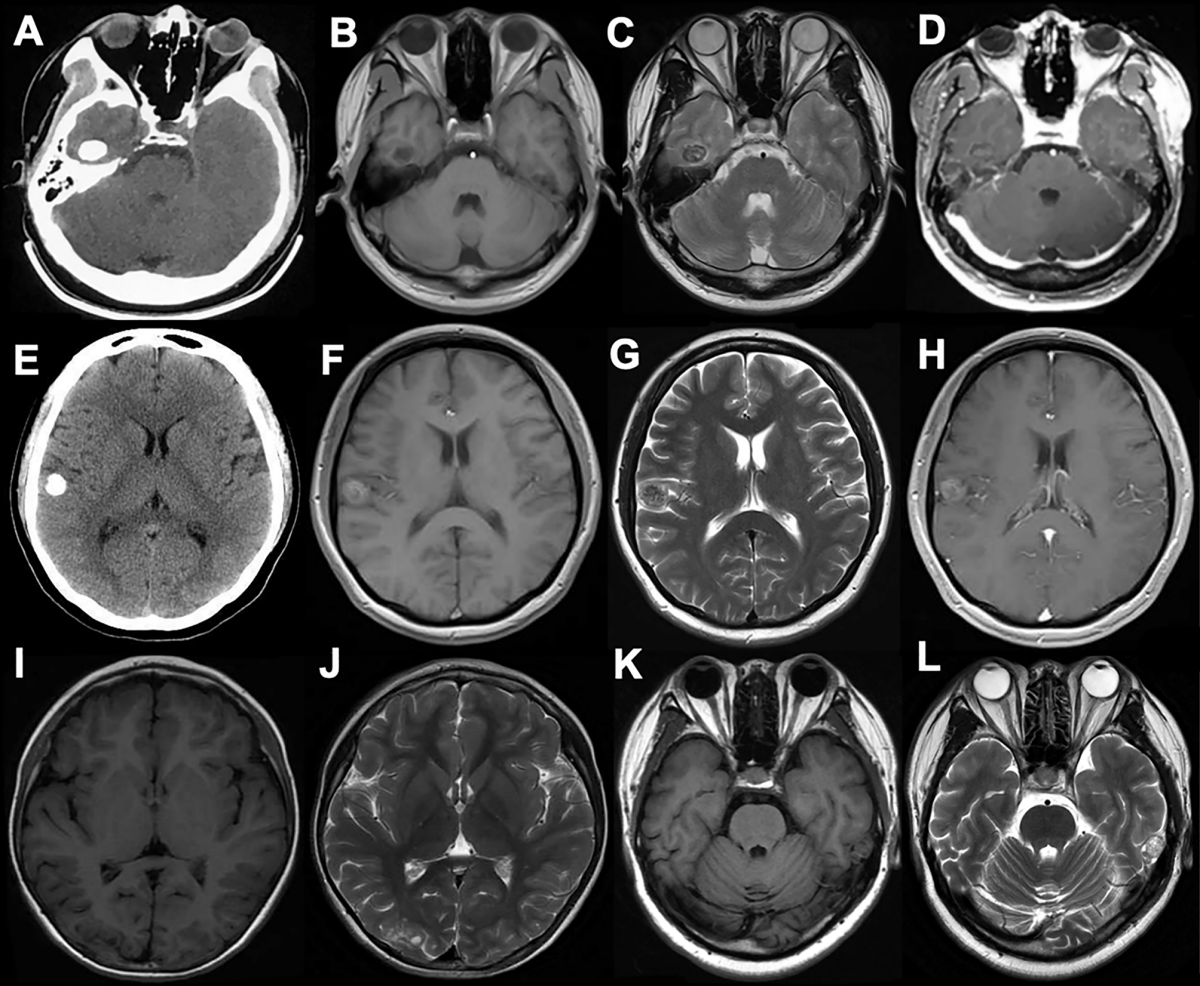
The CT, MRI manifestation of PLNTY. Dense calcifications were often demonstrated in CT images **(A, E)**. The MRI scans often appeared as iso- or low signal in T1W1 **(B, I, K),** and also can be slight hyperintense because of the composition of calcium salts **(F)**. The increased or mixed-signal were seen in T2W2 **(C, G, J, L)**, and the cystic components were also seen in **(J, L)**. No enhancements were shown in post-gadolinium T1-weighted MR images **(D, H)**.

The lesion locations were temporal lobe in 4 (50%) patients, frontal lobe in 3 (37.5%) patients, and occipital lobe in 1 (12.5%) patient. Among the 4 PLNTY located in the temporal lobe, none was in the mesial temporal lobe. Three lesions were located on the left side, and five lesions were located on the right side. Except in 3 cases (2 located in the frontal lobe, 1 located in the temporal lobe), all lesions were superficially situated (cortical/subcortical location). The CT imaging showed a lesion-associated calcification in 6 cases (75%), while a cystic component in 4 cases (50%). A well-delineated region was present in all cases in MRI images. Save for two cases (d, e), the tumor manifested slightly hyperintensity in T1WI, all lesions demonstrated iso- or hypointensity in T1WI. Six cases demonstrated increased or mixed-signal on T2WI and 3 lesions manifested slight or partial enhancement in post-gadolinium T1WI. Only case d was associated with a slight mass effect and edema (**Figure 1**).

### Surgical Resection and Seizure Outcomes

**Figure 2:**
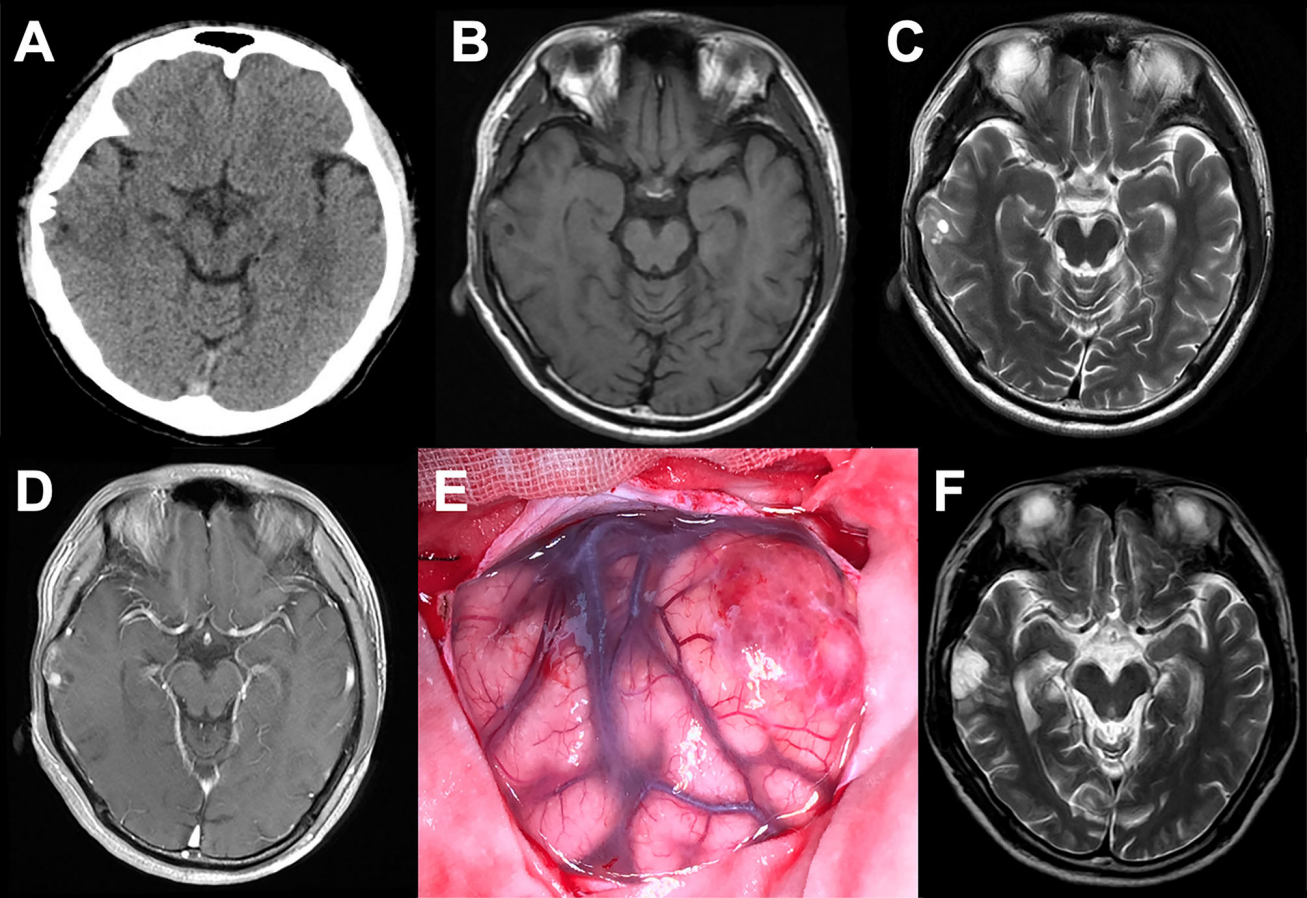
Data of case c. **(A)** Axial CT showing a superficial situated mass in the right temporal lobe with patchy calcification **(B–D).** Preoperative MR scans. The mass displayed a heterogeneous signal with cystic components, and a hypointense signal on T1WI, a hyperintense signal on T2WI, and partial enhancement on T1W1 post-gadolinium imaging. **(E)** Operative view before tumor resection. **(F)** Postoperative MRI showing the gross-total resection. The patient was seizure-free for 22 months.

The ER occurred in 2 (25%) cases, with lesions being located in the temporal lobe and occipital lobe respectively, and the other 6 (75%) cases achieved GTR. Both PR and STR were absent in this study. All patients recovered well postoperatively without immediate complication. The follow-up duration ranged between 11 and 42 months, with an average of 27 months, and no recurrence was observed in all the cases. Six patients (75%) were completely seizure-free (Engel class I) with or without AEDs, and the Engel class I of ER and GTR were achieved in 2 cases (100%) and 4 cases (66.7%) respectively (**Table 1** and **Figure 2**).

**Table 1:** Clinical, radiologic, and pathological characteristics of 8 PLNTY cases.

Case	Gender/Age (years)	Symptoms/Duration (months)	Tumor location	Calcification	Cystic component	T1, T2 and enhancement signals	Treatment	Immunohistochemistry					1p/19q co-deletion	BRAF mutation	Seizure outcome
								GFAP	Olig-2	ATRX	P53	Ki-67			
a	M/5	Epilepsy/37	Right occipital	No	Yes	T1: iso; T2: mixed hyper; En: none	ER	(+)	(+)	(+)	(−)	+2%	none	Neg	Engel class I
b	M/25	Epilepsy/3	Right temporal	Yes	No	T1: hypo; T2: mixed hypo; En: none	ER	(+)	(+)	(+)	(−)	<+2%	none	BRAF V600E	Engel class I
c	M/21	Epilepsy/50	Right temporal	Yes	Yes	T1: iso; T2: mixed hyper; En: partial	GTR	(+)	(+)	(+)	Weakly+	3%	none	BRAF V600E	Engel class I
d	M/51	Epilepsy/6	Left frontal	Yes	No	T1: hyper; T2: hyper; En: slight	GTR	(+)	(+)	(+)	(−)	+2%	none	Neg	Engel class I
e	F/30	Epilepsy/2	Right frontal	Yes	No	T1: hyper; T2: mixed hypo; En: none	GTR	(+)	(+)	(+)	(−)	+2%	none	N/A	Engel class I
f	M/46	Epilepsy/24	Left temporal	Yes	Yes	T1: hypo; T2: hyper; En: none	GTR	(+)	(+)	(+)	(−)	1%	none	BRAF V600E	Engel class II
g	M/28	Epilepsy/10	Left temporal	Yes	No	T1: hypo; T2: hyper; En: none	GTR	(+)	(+)	(+)	(−)	+<1%	none	N/A	Engel class I
h	M/47	Epilepsy/7	Right frontal	No	Yes	T1: hypo; T2: hyper; En: slight	GTR	(+)	(+)	(+)	(−)	+5%	none	BRAF V600E	Engel class II

En, enhancement; ER, enlarged resection; GTR, gross total resection.

### Pathological Examination

**Figure 3:**
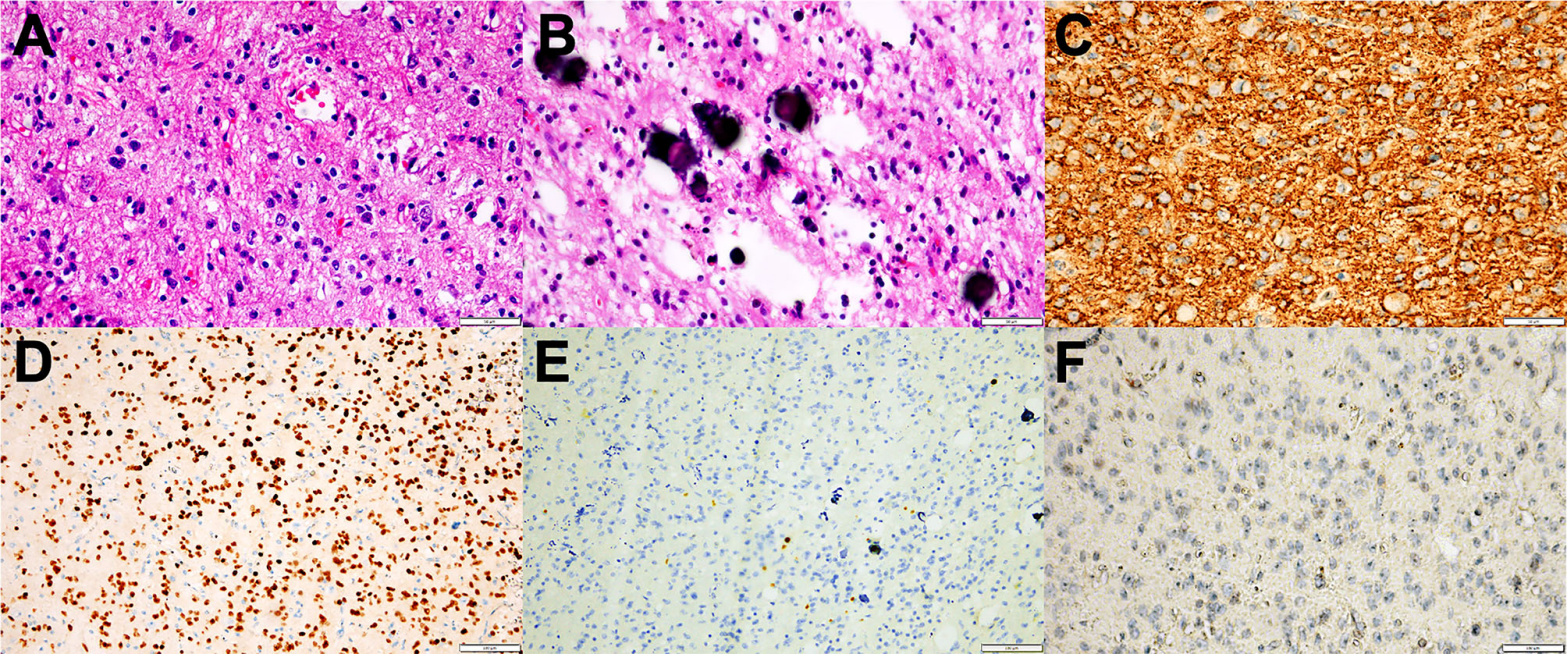
Representative morphological and immunohistochemical features of PLNTY (Case c and d). **(A)** H&E staining images showing oligodendroglioma-like cellular components with round or oval nuclei, abundant thin-walled blood vessels between tumor cells, and absence of mitoses and necrosis. **(B)** Extensive calcifications were shown in H&E staining. **(C)** Strong and widespread expression of CD34 in tumor cells. **(D)** Olig-2 immunostaining was positive in all cases. **(E)** Ki-67 labeling was below 2%. **(F)** IDH1 mutation negativity was demonstrated.

All tumors represented oligodendroglioma-like components arranged in an infiltrative manner with polymorphic cellular elements including pleomorphic and spindle cells, and have a round or oval nucleus with conspicuous perinuclear halo effects. Extensive microcalcification was identified in 6 of 8 cases. There were no typical elements of low-grade neuroepithelial neoplasms in all cases, such as myxoid microcysts, Rosenthal fibers, eosinophilic granular bodies, dysmorphic neuronal/ganglion cell forms, and neurocytic or ependymal rosettes. Peripheral focal cortex dysplasia (FCD) and neuronal vascular degeneration were present in case a who had preoperative drug-refractory epilepsy. The tumor necrosis and mitosis were absent in all cases (**Figure 3**).

**Figure 4:**
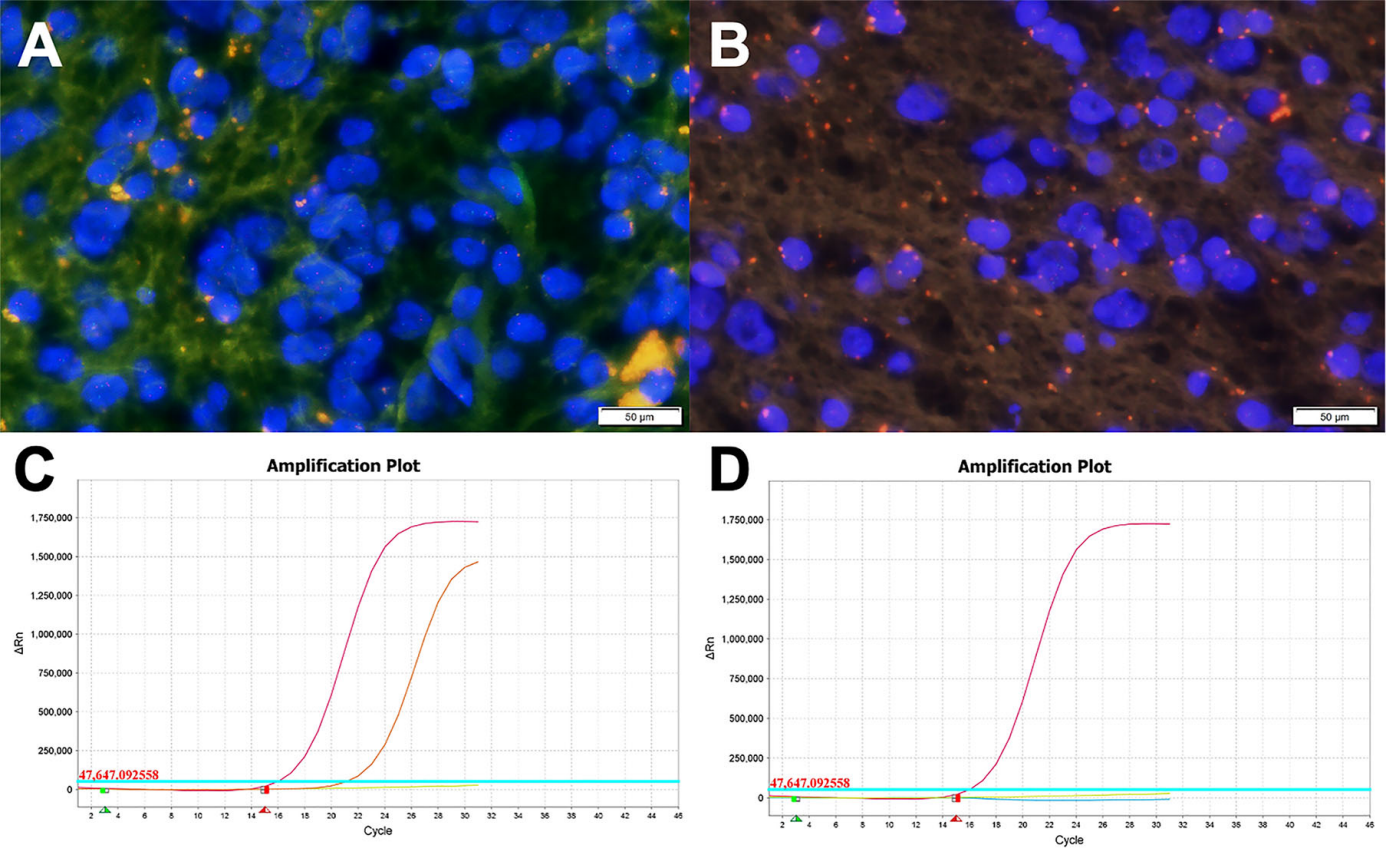
Molecular examination images of PLNTY. **(A, B)** FISH analysis for 1p/19q showing absence of chromosome 1p/19q deletion (Case c). **(C)** The BRAF V600E gene mutation was detected by the Real-Time PCR technique. The orange curve, the red curve, the green line, and the blue line represented the tested sample, the positive control, the negative control, and the threshold line respectively. The FAM signal, as seen in Case c, had an obvious amplification curve, and the CT value is less than 28, which means the BRAF V600E mutation is positive. **(D)** The BRAF V600E gene mutation of Case a (the blue line representing the tested sample). The test result was negative for the BRAF V600E gene mutation as the FAM signal had no amplification curve.

The patchy or widespread tumor cell expression of CD34 was demonstrated in all cases. Diffused positivity of Olig-2, expression of GAFP and ATRX were also manifested in 8 cases. Except for case c, which was focally and weakly positive for P53, the other cases had no overexpression of P53. The Ki-67 labeling index was below 2% in 6 cases, while that of case c and case h rose to 3 and 5%, respectively. IDH1 or IDH2 mutation and 1p/19q chromosomal codeletion were negative in all tumors. Six of the eight tumors in this study had qualified material for BRAF V600E mutations analyses, and BRAF V600E mutation was identified in four of six cases (66.7%) (**Figure 4**).

## Discussion

PLNTY is a recently recognized low-grade neuroepithelial tumor with a distinct biological entity affecting children and young adults. The age of the 10 patients initially reported by Huse et al. ranged from 4 to 32 years, with an average age of 17.6 years (1). With the increasing reports of PLNTY, the age spectrum has expanded, with the maximum age reported as 57 years old (21). The age of 55 patients reported in the current literature ranged from 4 to 57 years, with an average age of 20 years and a median age of 16 years. The age range of 8 patients in this study was 5–51 years old, with an average age of 31.6 years, which was older than that reported in the previous literature. It may be related to the fact that our center is mainly adult patients and the sample size is small. Like other LEATs, PLNTY can cause an early onset of epilepsy or a long history of refractory epilepsy because of its strong epileptogenic propensity (22). Of the 55 patients previously reported, 45 cases provided a clear medical history, and 37 of them had epilepsy onset (82.2%). In our study, 8 patients were all found to have intracranial lesions due to preoperative seizure, which is concordant with previous reports. The location of PLNTY also has the characteristics of LEATs, mainly in the temporal lobe (38/55 from the reports and 4/8 in our study), while the location in the parietal, frontal, and occipital lobes has also been reported.

The radiological characteristics of PLNTY have been gradually recognized, such as well-circumscribed, heterogeneous intralesional signal, and peripheral cysts. Moreover, the calcification on CT images is its more prominent feature. These calcifications are mainly the pattern of prominent central calcification or a few lamellar calcifications located in the periphery of the lesion. Three of the 10 cases reported by Huse et al. had complete images, and all of them had obvious circular calcifications on CT (1). Of the 9 cases reported by Johnson et al. (4), 8 cases had calcifications on CT. Five of 6 cases with calcification in this cohort were densely circular calcification foci, while the remaining was lamellar calcifications. PLNTY has the characteristics of benign mixed neuronal–glial tumor on MRI, however heterogeneous. It often manifests a well-circumscribed or slightly blurred lesion and hyperintense, isointense, or mixed intensity on T1 and T2 images. Moreover, many PLNTY often exhibits cystic components. The heterogeneous signal of PLNTY on MRI is mainly due to tumor calcification, while the signal of calcification is variable on MRI and is related to the composition of calcium salts. The typical imaging of PLNTY on T2WI demonstrates increased signal, mixed with the granulate mixed signals, which is called a “salt and pepper sign” in some research (5). Like the features of DNET or GG, PLNTY is seldom associated with significant mass effect or edema on MRI, while the tumor and its periphery often show hyperintense on T2-Flair images. PLNTY manifests slight or no enhancement on post-gadolinium T1WI, which indicates it has the characteristics of a benign tumor.

The pathological features of PLNTY were also gradually identified, namely, polymorphous appearance, the presence of oligodendroglioma-like cellular components, infiltrative growth pattern, and intense CD34 immunopositivity. Calcification is common in PLNTY, and the components of spindle, fibrous astrocytes and ependymoma-like pseudochrysanthemum clusters were also can be observed. Tumor mitosis, necrosis, and microvascular proliferation are rare in PLNTY. Eosinophilic granular bodies, myxoid microcysts, dysmorphic neuronal/ganglion cell forms, neurocytic or ependymal rosettes, and other characteristic structures were also absent in PLNTY, which can differentiate it from GG, DNET, PXA, and pilocytic astrocytoma. Intense and widespread tumor cell expression of CD34 is an immunohistochemical characteristic of PLNTY, however no specificity. The expression of CD34 can be observed in some LEATs, such as GG, DNET, and astrocytoma, and is also identified in regional cortical dysplasia which is associated with FCD. This is also the reason why this type of tumor has dual pathology and secondary epilepsy (23). On the other hand, the expression of CD34 is a useful tool that can readily discriminate PLNTY and oligodendroglioma, as CD34 is present in PLNTY and absent in oligodendroglioma (2, 24). Except for the expression of CD34, GAFP positivity is usually seen in PLNTY and Olig-2, which indicates the neuroepithelial origin of the tumor and the oligodendroglioma-like components within the tumor. Additional immunohistochemical studies showed PLNTY demonstrated retained expression of ATRX. Most of the Ki67 labeling index of PLNTY was less than 2%, and a few can reach 3 to 5%. In our study, all cases were positive for CD34, GFAP, Olig-2, and ATRX. Except for the Ki67 index of case c and case h which are 3 and 5% respectively, the other Ki67 indexes are around 1–2%.

In addition, 1p/19q codeletion and IDH1 or IDH2 mutation are typical molecular features in oligodendroglioma. However, PLNTY often exhibits no chromosome 1p/19q codeletion and absence of IDH1 or IDH2 genes. In our study, immunohistochemical examination showed IDH1 or IDH2 mutation was absent in all cases, and the lack of 1p/19q codeletion was also detected in all cases by FISH test. According to the genetic alterations of PLNTY, BRAF 600E mutation and FGFR2/FGFR3 fusions have been recurrently described, however mutually exclusive (1, 3). They have been both confirmed to activate the mitogen-activated protein (MAP) kinase pathway which promotes unlimited cell division and proliferation, leading to tumor formation (7, 25, 26). BRAF 600E mutation was identified as a common cancer-associated mutation that can be observed in many other LEATs, namely, GG, DNET, and PXA (27, 28). Among 55 cases of PLNTY reported to date, 19 cases displayed BRAF mutation (34.5%), and FGFR2/FGFR3 fusions were exhibited in 23 cases (41.8%). Six of the 8 cases in our sample set had qualified specimens to identify BRAF V600E mutation, and 4 (66.7%) cases were found to be positive. Unfortunately, FGFR gene fusion was not detected in our study.

PLNTY has been described as a benign tumor, so the PLNTY patients without epilepsy can be followed up, and close follow-up is necessary as the malignant transformation of PLNTY has been reported (13). On the other side, surgical treatment is recommended for patients with PLNTY complicated with epilepsy, especially drug-refractory epilepsy, because its secondary epilepsy can deteriorate the quality of life of patients. In this context, surgical tumor resection of PLNTY may provide effective seizure control, and it appears to be well controlled by gross total resection. However, some patients with PLNTY still have seizures after tumor resection (1). This was also confirmed in our study, in which 2 of 6 (33.4%) patients who underwent GTR still had seizures after surgery. We think it may be related to the residual FCD. Although tumors of PLNTY were removed, the remaining peripheral FCDs were still epileptic. Therefore, gross total resection of the tumor may not be enough, and enlarged resection of the tumor with the epileptic zone (EZ) is required. FCD is known to coexist with LEATs (29), and nowadays, an increasing number of studies reported the coexistence of PLNTY and FCD (5, 8, 30). The double pathology of PLNTY may be more frequent in cases with long seizure duration and drug-refractory epilepsy (30). In our study, the peritumoral cortex was obtained in 2 patients (cases a, b) with ER, and peritumoral FCD was observed in case a with drug-refractory epilepsy, which was located in the occipital lobe. The patient was postoperative seizure-free at 27 months. On the other hand, the rate of Engel class I of ER was high than that of GTR. The significance, however, needs to be verified by follow-up studies with large samples.

Under the circumstances, the identification of the EZ is critical to postoperative seizure control. Stereotactic EEG (SEEG) may be an ideal choice to accurately identify the EZ, however will greatly increase the hospitalization costs of patients in clinical reality. Referring to our experience in other LEATs surgery, preoperative PET-MR image fusion can be utilized to observe the tumor and the surrounding low FDG uptake area, while surgical resection of these low FDG uptake areas may effectively improve the postoperative seizure-free rate. Certainly, further studies are necessary to determine its benefit in PLNTY surgery. In addition, intraoperative ECoG can also help to identify the EZ, as the application of intraoperative ECoG can significantly improve the outcome of seizure control (31, 32).

## Conclusion

PLNTY is a newly described low-grade neuroepithelial tumor which is often encountered in children and young adults. However, there are no specific diagnostic criteria of PLNTY because of the overlap of clinical, radiologic, histopathologic, and molecular characteristics between PLNTY and low-grade glioma or other mixed neuronal–glial tumors (2). These features, therefore, need to be combined to consider the diagnosis of PLNTY. PLNTY has high epileptogenicity, and its secondary epilepsy can deteriorate the quality of life of patients. Therefore, early surgical interventions are recommended for patients with PLNTY complicated with epilepsy. The identification of the epileptic zone is critical for the surgery, and enlarged resection may be a good prognosis factor for postoperative seizure-free.

## Data Availability Statement

The original contributions presented in the study are included in the article/supplementary material. Further inquiries can be directed to the corresponding author.

## Author Contributions

XRF, YY, JZ, WW, XK, RBQ, and CSN contributed to the conception and design of the study. XRF and YY organized the database. XRF performed the statistical analysis. XRF wrote the first draft of the manuscript. XRF, XK, and YY wrote sections of the manuscript. All authors listed have made a substantial, direct, and intellectual contribution to the work and approved it for publication.

## Conflict of Interest

The authors declare that the research was conducted in the absence of any commercial or financial relationships that could be construed as a potential conflict of interest.

## Publisher’s Note

All claims expressed in this article are solely those of the authors and do not necessarily represent those of their affiliated organizations, or those of the publisher, the editors and the reviewers. Any product that may be evaluated in this article, or claim that may be made by its manufacturer, is not guaranteed or endorsed by the publisher.
